# Results of Adrenalectomy for Isolated, Metachronous Metastasis of Breast Cancer: A Retrospective Cohort Study

**DOI:** 10.3389/fsurg.2021.671424

**Published:** 2021-06-09

**Authors:** Giulio Illuminati, Rocco Pasqua, Giuseppe D'Ermo, Marco Girolami, Bruna Cerbelli, Giulia D'Amati, Fabio Carboni, Enrico Fiori

**Affiliations:** ^1^Department of Surgical Sciences, University of Rome “La Sapienza”, Rome, Italy; ^2^Department of Surgery, “Pietro Valdoni”, University of Rome “La Sapienza”, Rome, Italy; ^3^Department of Medical-Surgical Sciences, Biotechnologies and Pathology, University of Rome “La Sapienza”, Rome, Italy; ^4^Department of Surgery, Regina Elena Cancer Institute, Rome, Italy

**Keywords:** adrenal metastases, breast cancer, adrenalectomy, open surgery, laparoscopic access

## Abstract

**Background and Aim:** Metachronous, isolated adrenal metastases from breast cancer are extremely rare. The aim of this study was to evaluate the results of adrenalectomy as a treatment of this uncommon condition.

**Methods:** Twelve female patients (median age: 68 years) underwent 13 adrenalectomies for isolated, metachronous metastases of breast cancer. Ten resections were performed thorugh open surgery and two were preformed through a laparoscopic approach. As main study endpoints, postoperative mortality, postoperative morbidity and disease-free survival were considered. Median length of follow-up was 40 months.

**Results:** Postoperative mortality was absent. Postoperative morbidity was 17%: one patient presented a postoperative pneumothorax requiring drainage and one patient required re-hospitalization 8 days after contralateral adrenalectomy for electrolyte imbalance. Two patients died of recurrent metastatic disease, 28 and 33 months respectively after adrenalectomy. One patient remained alive with hepatic metastases at 32 months from resection of adrenal recurrence. All in all, disease-free survival at 48 months was 75%.

**Conclusions:** Adrenalectomy for metachronous, isolated metastases of breast cancer can be performed with no postoperative mortality and minimal postoperative morbidity, enabling good long-term disease-free survival.

## Introduction

The adrenal gland is a relatively frequent site of metastasis due to its rich sinusoidal blood supply (1, 2). Metastases most often occur from lung, renal and gastrointestinal cancer (3–7), with squamous cell carcinoma and adenocarcinoma being the most commonly encountered histological diagnoses (1). In contrast, adrenal metastases from breast cancer account for only 2.9% of all adrenal metastases and are most often associated with synchronous metastases at other extra-adrenal sites (8). Furthermore, distant visceral metastases could be markers of aggressive neoplastic spread and would suggest a need for adjuvant or palliative treatment rather than aggressive surgical resection. In selected cases, however, resection of an isolated adrenal metastasis may lead to long-term survival (3–7). It was with this assumption in mind that we first prospectively performed adrenalectomy whenever a metachronous, isolated, metastasis from prior breast cancer was suspected and then, wishing to verify the validity of our hypothesis, we retrospectively reviewed the results of this treatment.

## Patients and Methods

From January 2000 to May 2020, 12 consecutive female patients with a median age of 68 years (range, 28–77 years) underwent 13 adrenal resections for metachronous, isolated and single metastases from breast carcinoma, at an academic, tertiary care hospital and a regional, tertiary care oncological hospital, following treatment of the primary tumor at other institutions. The 13 resections represent 3.3% of the 368 adrenalectomies performed for neoplasms at the two centers during the same study period. The catchment area was a capital city with 4 million inhabitants. Metachronous metastasis was defined as distant tumor recurrence three months or more after treatment of the primary cancer. Patient data were reported in accordance with the Strobe statement for observational studies (9). During the study period, no patient other than those included in the study was assessed for isolated, metachronous adrenal metastasis of breast cancer and denied operation. This translates to a 100% acceptance rate.

One patient underwent a contrateral adrenalectomy for metachronous metastases 15 months after a prior adrenalectomy for the same metastatic disease.

The stage of the primary disease at the time of the index treatment was stage II (nine patients) or stage III (three patients), and histology consisted in invasive ductal carcinoma (IDC) (eight patients) and invasive lobular carcinoma (ILC) (four patients). The initial surgical treatment consisted of a quadrantectomy associated with an axillary node dissection in the nine stage II, patients and a mastectomy with node dissection in the three stage III patients.

**Figure 1:**
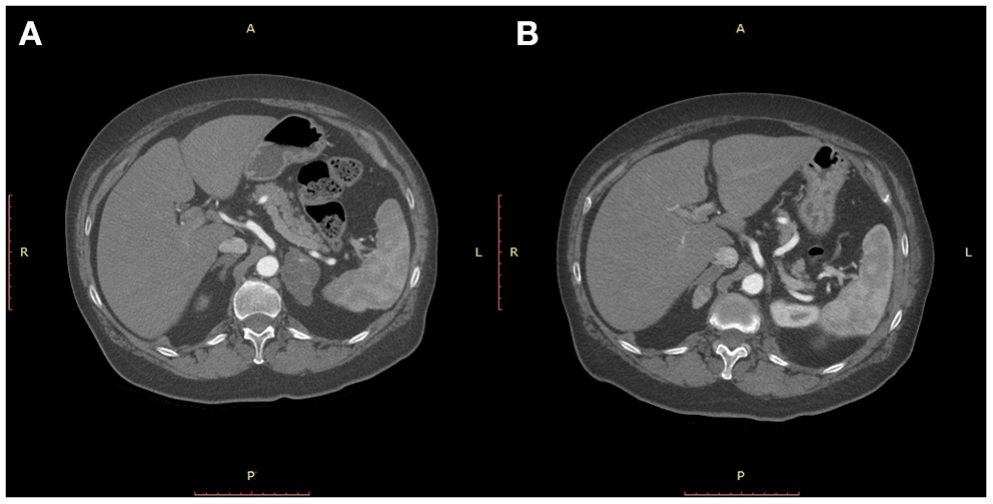
CT-scan of the abdomen showing a 5 cm diameter mass of the left adrenal gland 37 months after a quadrantectomy for an infiltrating ductal carcinoma of the breast **(A)** and a metachronous 2 cm diameter mass of the right adrenal 15 months after left adrenalectomy **(B)**.

The adrenal masses were initially diagnosed in the ultrasound studies of the abdomen performed as part of oncologic follow-up protocols. Preoperative work-up for all patients included an ultrasound study of the abdomen and a CT-scan of chest, abdomen and pelvis and bone scintigraphy, the objective being to detect possibly asymptomatic or unknown synchronous metastases elsewhere. The two most recent two patients of the series, who were examined subsequent to January 2018, also underwent as part of their preoperative workup a ^18^F-FDG positron emission tomography (PET) scan, the objective being to definitively rule out any secondary metastatic location of the disease other than the adrenal gland. Preoperative biopsy was not performed, the assumption being that compared to the at times inconclusive results of needle biopsies, surgical resection, would be diagnostic and curative at the same time. None of the patients presented signs and symptoms of adrenal insufficiency. All of the patients underwent a thorough pre-operative functional study with plasma levels of ACTH and Ca 15-3, urinary vanillylmandelic acid output/24 h together with catecholamines and their metabolites concentration in urine and plasma and bone scintigram to rule out primary adrenal tumors and other synchronous extra-adrenal localizations. Potential aldosterone overproduction was not assessed. Radiological features confirming the metastatic nature of the adrenal mass at CT-scan consisted of a well-delimited, homogeneous low density nodule, without aspects of the hemorrhage, calcifications and necrosis that are usually typical of primary, functioning adrenal tumors. These findings coupled with the patients' past history and the results of the above-mentioned work-up prompted indication for surgical resection of the mass. Indication for surgery was given in a multidisciplinary approach including surgeons and also the oncologists caring for the patient. Written consent for operation was obtained from all patients, and due to the retrospective nature of the study, institutional approval was waived. Ten patients underwent open surgical adrenalectomy. One patient underwent laparoscopic adrenalectomy for a 3 cm diameter metastasis, whereas the one patient with bilateral, metachronous disease underwent a prior open adrenalectomy for a 5 cm diameter metastasis, followed by a laparoscopic adrenalectomy, 15 months later for a 2 cm diameter secondary disease (Figure 1). In this patient, estradiol, progesterone and P53 receptors in both primary tumor and metastases were positive.

Open resection was performed using a retroperitoneal approach via a lombotomy over the 11^th^ rib with partial resection of the rib itself and care taken to avoid entering the pleural space. Laparoscopic resection was performed using a 4-trocar transperitoneal approach.

Concerning surgical access, due to a high number of cases in the early part of the series, mini-incisions by lombotomy, which was in wide use at that time, was performed more frequently than laparoscopy. At the present, by contrast, due to progressive and pronounced improvement of laparoscopic skills, laparoscopic access is preferred for all adrenal lesions not exceeding a diameter of 5 cm.

Postoperatively, the patients were referred to the oncology unit of each center participating in the study for possible adjuvant treatment and follow-up. Controls were carried out according to standard oncological protocols, which included an ultrasound study of the abdomen every 6 months, a chest X-ray every 12 months, laboratory assessment of adrenal function every 3 months and laboratory assessment of tumor markers (CA 15-3, Ca-125 and CEA) every 6 months.

### The Study's Specific Definitions and Analyses

As the main endpoints of the study, postoperative mortality, postoperative morbidity and disease-free survival were considered. Postoperative mortality was defined as any death occurring within 30 days from operation or during the whole length of hospitalization for surgical treatment of the metastasis. Postoperative morbidity was defined as any complication following operation requiring either reintervention or any surgery-related additional treatment. Postoperative mortality and morbidity were evaluated according to the Clavien-Dindo classification (10). Disease-free survival was defined as overall survival minus any death related to the disease or any recurrence of the disease, evidenced either clinically or in imaging protocols applied during follow-up.

### Statistics

Non categorical variables were expressed as medians and inter-quartile range (IQR). Disease-specific survival was assessed with the life-table analysis and outlined with Kaplan-Meier curves.

## Results

**Figure 2:**
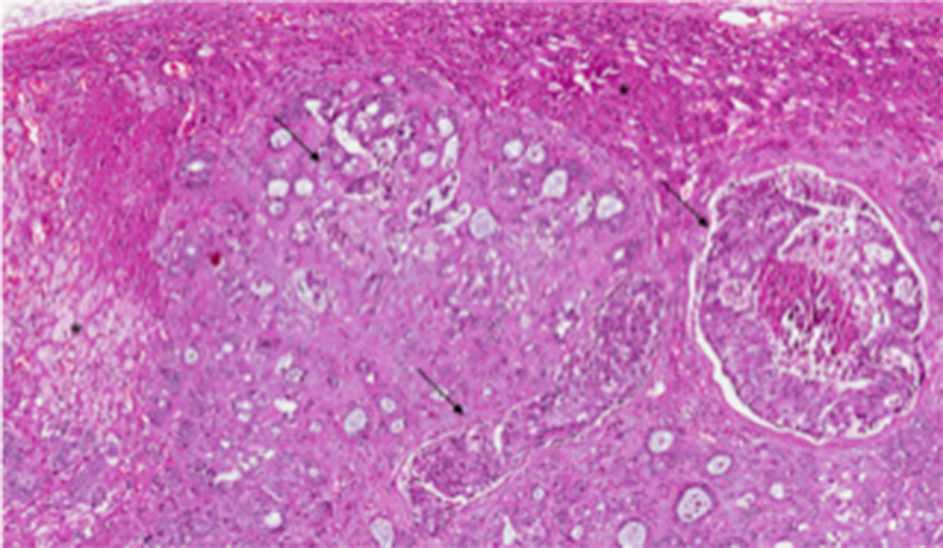
Pathological examination of adrenal metastatic disease. Histological examination revealed metastasis from invasive breast carcinoma, arranged in solid nests or ductal structures (black arrowheads) in adrenal gland (black asterisks) (hematoxylin and eosin, original magnification 4×).

The median body mass index (BMI) of the patient population was 21.7 Kg/m^2^ (IQR, 20.25–22.8 Kg/m^2^). The median interval between treatment of the primary tumor and detection of adrenal metastasis was 49 months (IQR, 34–63.5 months). The median diameter of the metastatic tumor was 3 cm (IQR, 2.5–5 cm) and origin from the breast was confirmed at pathological examination of the resected specimen (Figure 2). The median length of follow-up was 40 months (IQR, 22.4–74.5 months). The essential clinical data of the patients are reported in Table 1. An R0 resection margin could be obtained in all cases. Histology and immunohistochemistry of the metastases were superposable to those of primary tumor.

**Table 1:** Main clinical data of the patient population.

**Patient number**	**Age (years)**	**BMI (Kg/m^**2**^)**	**Diameter of the mass (cm)**	**Histology**	**Follow up (months)**	**Outcome**
1	61	23	4	IDC	77	Alive, free from disease
2	68	20.1	3	ILC	28	Dead, osseous metastases
3	54	19.7	2	ILC	33	Dead, hepatic and osseous, metastases
4	42	20.4	5	IDC	72	Alive, free from disease disease
5	69	21.7	3	IDC	64	Alive, free from disease disease
6	76	24.2	6	ILC	23	Alive, free from disease
7	36	20.9	7	IDC	89	Alive, free from disease
8	28	22.3	4	ILC	32	Dead, hepatic metastases
9	47	25.8	3	IDC	108	Alive, free from disease
10	79	21.6	2	IDC	41	Alive, free from disease
11	77	20.4	3	IDC	40	Alive, free form disease
12	70	22.6	5	IDC	15	Alive, free from disease
13	70	22.6	2	IDC	2	Alive, free from disease

*IDC, invasive ductal carcinoma; ILC, invasive lobular carcinoma; BMI, body mass index*.

*Numbers 12 and 13 refer to the same patient operated of bilateral adrenalectomy*.

Seven patients with IDC and two patients with ILC presented positive estrogen receptors with Ki 67 > 20%, and received postoperative, tamoxifen-based hormonal therapy after index resection of the metastatic tumor. The four patients with ILC and two patients with IDC also underwent adjuvant chemotherapy. One patient with HER2+ receptors on the adrenal metastasis bearing an IDC received postoperative therapy with trastuzumab. HER2 status on the primary tumor was not available on admission for the treatment of the adrenal mass.

### Main Endpoints

**Figure 3:**
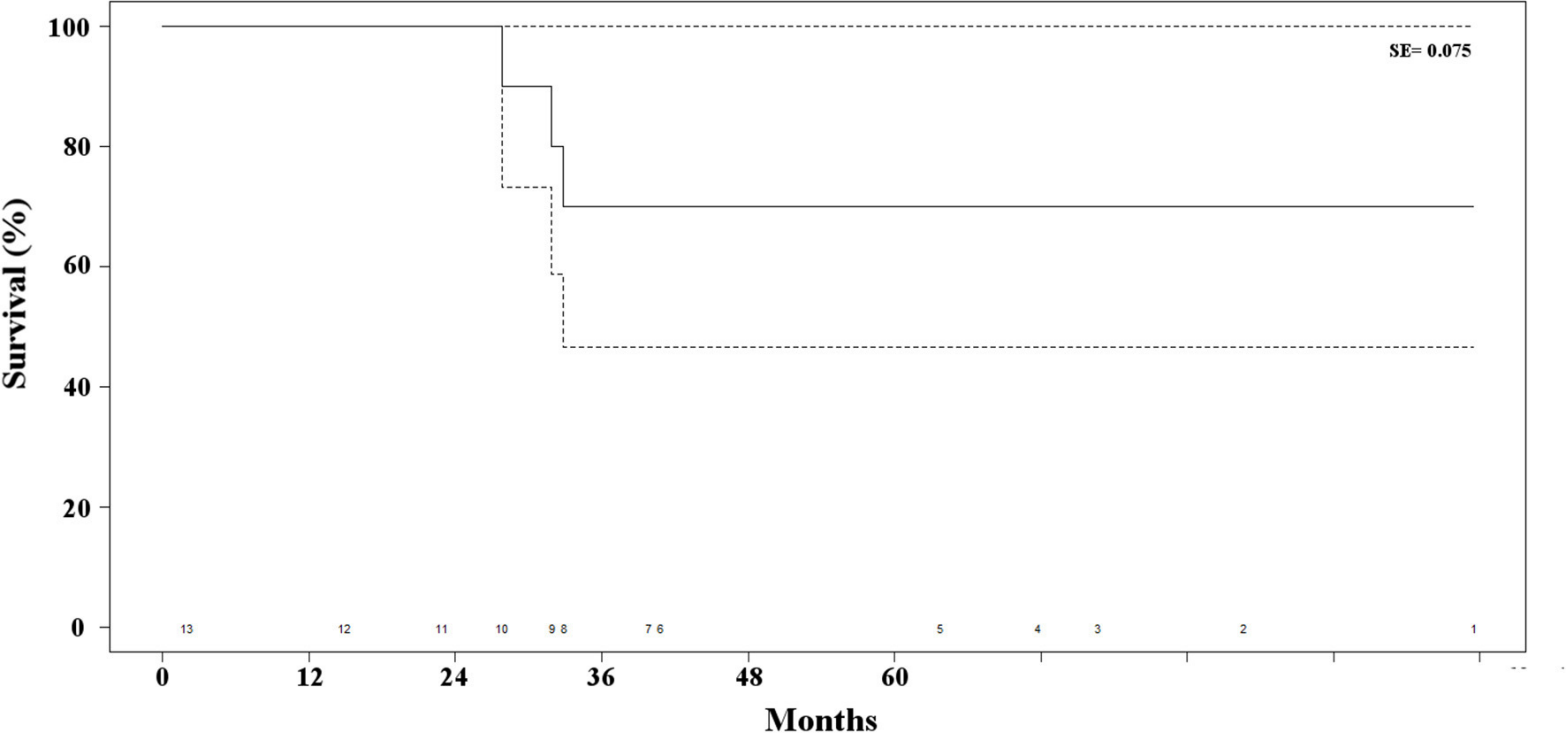
Kaplan-Meier estimate of survival of the patients' series. The dotted lines represent the standard error (SE) and the numbers at the bottom of the graphic the number of patients at risk for each time interval.

Postoperative mortality was absent in this series. One patient undergoing open adrenalectomy required drainage of postoperative pneumothorax, due to inadvertent entering of the pleural cavity. One additional patient required re-hospitalization eight days after contralateral adrenalectomy for electrolyte imbalance, which could be medically managed with success. All in all, the postopeartive morbidity rate was 17% with one grade II complication (8.5%) and one grade IIIa complication (8.5%) according to the previously cited classification (10). The median length of postoperative hospital stay was 7 days (IQR, 6–9 days). Two patients died of bone and hepatic metastases, 28 and 33 months, respectively, after adrenalectomy. One patient remained alive, but with hepatic metastases 32 months after resection of the adrenal recurrence. All of these patients were suffered by ILC (*p* = 0.03). All in all, disease-free survival at 48 months was 75% (Figure 3). As no other patient died of any cause other than progression of the disease during follow-up, overall and disease-free survival were superposable. The main results of the series are reported in Table 2.

**Table 2:** Main study's endpoints.

**Endpoint**	**Measure**
Postoperative mortality (*n*, %)	—
Postoperative morbidity (*n*, %)	2, 14
Postoperative lenght of stay (days)	7 (range, 5–13)
Disease-free survival at 48 months (%)	75

## Discussion

The results of this study suggest that aggressive resection of isolated adrenal metastases from breast cancer associated with adjuvant treatment allows excellent disease-free survival with minimal surgical morbidity and no surgery-related mortality. This statement, however, may not apply to ILC, given its aggressive clinical course, which could advice an adjuvant treatment as a valid alternative.

While adrenal metastases from different organs and different histological type of cancers are well-known and frequent, isolated metastases from breast cancer are very rare and the knowledge of their biological behavior is limited to a few isolated case reports (1, 8). Among different histologic types, invasive ductal carcinoma, the most common of breast cancers, should be less likely to be associated with distant and especially adrenal metstases (8, 11–13), as opposed to invasive lobular carcinoma, which is less frequent but more aggressive (8). The results of the present series seem to show that in fact, both histologic types can be associated with metachronous adrenal metastases and that, in case of IDC, surgical resection can allow long-term survival. It also bears mentioning that the status of estrogen receptors at the time of the index procedure did not predict any particular likelihood of metachronous adrenal metastases, and negative receptors for estradiol, progesterone and P53 were not significantly correlated with the probability of adrenal metastases, although the only patient presenting a metachronous metastasis to the contralateral adrenal gland had positive estrogen receptors. However, due to the small number of included patients, no reliable prediction based on receptors can be drawn from this series. On the other hand, and as expected, invasive lobular carcinoma as a primary histology of the disease was associated with a much worse prognosis than invasive ductal carcinoma, as all three patients in the series presenting metachronous metastatic disease died a few months after adrenalectomy. Generally speaking, metastatic adrenal disease is less frequent in women than in men, and in the former gender breast cancer is even less frequent (1, 14, 15). Nonetheless, in this series, preoperative differentiation between benign, primary adrenal masses and possible metachronous breast cancer secondary localization was effective, based as it was on functional studies, and CT scan morphology. Adrenal adenomas usually appear as a low attenuation lesion, due to their intracellular lipid concentration, whereas non-calcified, non-hemorrhagic adrenal nodules with high pre-contrast attenuation are more likely to be secondary malignant localizations (16–18).

The size of primary adrenal masses is thought to be correlated with malignancy, with lesions of <4 cm diameter being considered most likely to be benign (16, 19–21), in the present series, the diameter of with metastatic nodules varied from 2 to 7 cm.

Progressive improvement of laparoscopic skills and techniques as lead to an increasing number of adrenalectomies performed laparoscopically, with either a transperitoneal or a retroperitoneal access. The transperitoneal route may allow a larger working space, whereas the retroperitoneal access bears the advantage of pre-existing anatomical planes and avoidance of handling of the bowel (22). Actually both accesses allow superposable results and are performed according to surgeon' familiarity and choice (23, 24).

### Limitations

The present study has three main limitations consisting of its retrospective nature, long time span and small population. However, the data were objectively collected and assessed. Hopefully, further larger and prospective studies will support the findings of the present one and the generalisability of its results.

## Conclusions

In conclusion, the results of the present study suggest that an aggressive attitude toward curative surgical resection of metachronous secondary adrenal masses after prior resection of primary breast cancer may be justified. Resection can be achieved with minimal surgical risk and may allow prolonged disease-free survival.

## Data Availability Statement

The raw data supporting the conclusions of this article will be made available by the authors, without undue reservation.

## Ethics Statement

Ethical review and approval was not required for the study on human participants in accordance with the local legislation and institutional requirements. The ethics committee waived the requirement of written informed consent for participation.

## Author Contributions

GI, GD'E, and EF: conception and design of the study. RP, MG, BC, GD'A, and FC: literature search, data collection, and extraction of relevant information. GI, FC, and EF: analysis and interpretation. GI: writing the article. GI, GD'E, FC, and EF: critical revision. All the Authors contributed to the article and approved the submitted version of the manuscript.

## Conflict of Interest

The authors declare that the research was conducted in the absence of any commercial or financial relationships that could be construed as a potential conflict of interest.
